# Adopting the Rumsfeld approach to understanding the action of levodopa and apomorphine in Parkinson’s disease

**DOI:** 10.1007/s00702-023-02655-0

**Published:** 2023-05-20

**Authors:** P. Jenner, C. Falup-Pecurariu, V. Leta, M. Verin, M. Auffret, Roongroj Bhidayasiri, D. Weiss, F. Borovečki, W. H. Jost

**Affiliations:** 1 Faculty of Life Sciences and Medicine, Institute of Pharmaceutical Sciences, King’s College London, London, SE1 1UL UK; 2 Department of Neurology, Transylvania University, 500036 Brasov, Romania; 3 Parkinson’s Foundation Center of Excellence at King’s College Hospital; Department of Basic and Clinical Neuroscience, The Maurice Wohl Clinical Neuroscience Institute, King’s College London and National Institute for Health Research (NIHR) Mental Health Biomedical Research Centre, Institute of Psychology, Psychiatry and Neurosciences, King’s College London, London, UK; 4 Institut des Neurosciences Cliniques de Rennes (INCR); Behavior and Basal Ganglia Research Unit, CIC-IT, CIC1414, Pontchaillou University Hospital and University of Rennes, Rennes, France; 5 France Développement Electronique (FDE), Monswiller, France; 6 Department of Medicine, Faculty of Medicine, Chulalongkorn Centre of Excellence for Parkinson’s Disease and Related Disorders, Chulalongkorn University and King Chulalongkorn Memorial Hospital, Thai Red Cross Society, Bangkok, 10330 Thailand; 7 The Academy of Science, The Royal Society of Thailand, Bangkok, 10330 Thailand; 8 Department for Neurodegenerative Diseases, Centre for Neurology, Hertie-Institute for Clinical Brain Research, Hoppe-Seyler-Str. 3, 72076 Tübingen, Germany; 9 Division for Neurodegenerative Diseases and Neurogenomics, Department of Neurology, University Hospital Centre Zagreb, 10000 Zagreb, Croatia; 10 Parkinson-Klinik Ortenau, Kreuzbergstr. 12-16, 77709 Wolfach, Germany

**Keywords:** Levodopa, Apomorphine, Parkinson’s disease, Mechanism of action, Future therapy

## Abstract

Dopaminergic therapies dominate the treatment of the motor and non-motor symptoms of Parkinson’s disease (PD) but there have been no major advances in therapy in many decades. Two of the oldest drugs used appear more effective than others—levodopa and apomorphine—but the reasons for this are seldom discussed and this may be one cause for a lack of progress. This short review questions current thinking on drug action and looks at whether adopting the philosophy of ex-US Secretary of State Donald Rumsfeld reveals ‘unknown’ aspects of the actions of levodopa and apomorphine that provide clues for a way forward. It appears that both levodopa and apomorphine have a more complex pharmacology than classical views would suggest. In addition, there are unexpected facets to the mechanisms through which levodopa acts that are either forgotten as ‘known unknowns’ or ignored as ‘unknown unknowns’. The conclusion reached is that we may not know as much as we think about drug action in PD and there is a case for looking beyond the obvious.

## Introduction

The treatment of Parkinson's disease (PD) remains dominated by the use of dopaminergic agents because of their undoubted effectiveness in improving the major motor and some non-motor symptoms of this disorder (Chaudhuri and Schapira 2009; Mao et al. 2020). Since the introduction of levodopa in the 1960s (Fahn 2008), we have seen the development of dopamine agonist compounds (including ergot based and non-ergot-based drugs), adjuncts to the use of levodopa (DDC, COMT and MAO-B inhibitors) and a move away from the classical oral administration used in PD (including intraduodenal administration, subcutaneous infusion and transdermal application) (Armstrong and Okun 2020a, b; Bloem et al. 2021; Kalia and Lang 2015; Poewe and Mahlknecht 2020; Poewe et al. 2017). However, it remains the case that despite these advances, levodopa is still considered to be the most effective compound for treating PD and recently there has been a return to using it as first-line therapy that reverses the later use, levodopa sparing and levodopa phobia concepts of the past (Agid et al. 1999; Katzenschlager and Lees 2002; Olanow 2019). Whilst the dopamine agonists provide an alternative strategy for therapy, none of the orally used dopamine agonist compounds (largely ropinirole and pramipexole) are attributed with the same efficacy as levodopa (Tolosa et al. 1998). In contrast, the intermittent subcutaneous injection of apomorphine and its subcutaneous infusion—a drug that lacks oral bioavailability—is considered by some as being as effective on acute and repeated administration as levodopa for reasons that we will explore later in this paper (see for example Castillo-Torres et al. 2023; Dewey et al. 2001).

The overall picture is that whilst advances have been made and more options for symptomatic treatment are available and adverse events have been addressed, there have not been the milestone developments of novel drug therapies that have supplanted the activity of the older drugs in clinical practise. We must ask ourselves why we have not seen the progress in symptom control that might have been expected from the research and development effort which has been put into producing novel ‘dopaminergic’ approaches to therapy. Why do apomorphine and levodopa stand out from other dopaminergic treatments in a way which has stood the test of time? We must ask whether we understand how these drugs work or are we being presumptive about the way in which they influence neuronal function to cause improvement in PD.

It is not the objective of this review to revisit the anatomy, physiology and pharmacology of the dopaminergic system or to reiterate the reasons for the changes in dopaminergic function which take place in PD and underlie the efficacy and adverse effects of current drug treatment (see Beaulieu and Gainetdinov 2011; Fuxe et al. 2015; Hassan and Thakar 1988; Kaasinen et al. 2021; Missale et al. 1998; Strange 1993). The object of this paper is to merely stimulate thought about the way in which apomorphine and levodopa produce their effects in PD and to that end we have turned to an unlikely source for inspiration namely the former US Secretary of State Donald Rumsfeld who was widely quoted with the following in relation to a question over the lack of evidence linking the government of Iraq with the supply of weapons of mass destruction to terrorist groups (There are unknown unknowns—Wikipedia):

*‘Reports that say that something hasn’t happened are always interesting to me, because as we know, there are known knowns; there are things we know we know. We also know there are known unknowns; that is to say, we know there are some things we do not know. But there are also unknown unknowns—the ones we don’t know we don’t know’*.

Rumsfeld had adopted the argument from one based on a philosophy widely used by intelligence agencies and in project management known as the Johari window. In a film based his life, Rumsfeld initially defines “unknown knowns” as “the things you think you know, that it turns out you did not”, and toward the end of the film, he defines the term as “things that you know, that you don't know you know” [Krogerus, Mikael (2012). *The Decision Book: Fifty Models for Strategic Thinking*. Roman Tschäppeler, Jenny Piening (1st American ed.). New York: W.W. Norton & Co. pp. 86–87; Girard, John; Girard, JoAnn *A Leader’s Guide to Knowledge Management: Drawing on the Past to Enhance Future Performance*. Business Expert Press. pp. 55]. Beyond these three categories, there is a fourth that has been added, ‘the unknown known, that which one intentionally refuses to acknowledge that one knows’.

Adopting the Rumsfeld matrix to look at drug action in PD, we will discuss the actions of apomorphine and levodopa and examine some of the apparently ‘known unknowns’ and ‘unknown unknowns’ that affect the actions of levodopa. The objectives are to initially refresh your knowledge of some aspects of dopamine receptor pharmacology, then to explore the pharmacology of apomorphine and levodopa and to suggest that there are ‘unknowns’ that explain why we do not fully understand how these drugs work. Perhaps importantly, we look at bits of the pharmacology of levodopa that usually get forgotten (‘the unknown known’) but which illustrate the incompleteness of our knowledge.

## Setting the dopaminergic scene in Parkinson’s disease

The first thing to emphasise is the complexity of PD not only from the perspective of its symptomatology but also from the diversity of the pathological and biochemical changes that take place both within the basal ganglia and throughout the rest of the brain and in the peripheral nervous system involving both dopaminergic and non-dopaminergic neuronal systems (Alexander 2004; Armstrong and Okun 2020b; Berg et al. 2021; Bloem et al. 2021; Dauer and Przedborski 2003; Jankovic and Tan 2020; Kalia and Lang 2015; Lees et al. 2009; MacMahon Copas et al. 2021; Poewe et al. 2017; Schapira et al. 2017; Titova et al. 2017). This complexity which underlies the motor and non-motor symptoms of PD highlights a need to restore neuronal function more widely in brain rather than limiting the pharmacological approaches to treatment to basal ganglia and to the loss of dopaminergic innervation (Lang and Obeso 2004).

However, it is the dopaminergic system to which we attribute most of the effects of drugs such as levodopa and apomorphine. Multiple dopamine receptor subtypes have been described, cloned and characterised and divided into the two main families—the D-1-like (D-1 and D-5 receptors) and the D-2-like (D-2, D-3 and D-4 receptors) dopamine receptors—and not surprisingly, dopamine interacts with all these receptor subtypes (Beaulieu et al. 2015). Although there has been an era of dopamine receptor biochemistry, pharmacology and behavioural analysis, there has been little exploitation of the specific role of the various subtypes in either neurology or psychiatry that has translated to man despite much preclinical endeavour (see Giorgioni et al. 2021; Kiss et al. 2021; Torrisi et al. 2023; Yang et al. 2020). In PD, there has been particular interest in utilising dopamine agonists that interact with D-2-like receptors (Ferraiolo and Hermans 2023; Juza et al. 2023)—as D-1-like receptors were in this era blamed for the adverse event profile of levodopa, notably dyskinesia (see Bastide et al. 2015). But even today, we have no drugs which selectively interact with the D-1-like family of receptors and through which there might be much to be gained as detailed below. We also seek to avoid the D-3 receptor as this has associations with the impulse control disorders common in dopamine agonist drug use and with dyskinesia expression or onset (Chagraoui et al. 2022; Lanza and Bishop 2021; Seeman 2015).

In PD, dopamine receptors are invariably considered in relation to basal ganglia function but in reality, there is a widespread distribution of dopamine receptors in cortical and sub-cortical brain areas with a differential or topographical distribution of subtypes within each of these regions [dopamine receptors (diff.org)] (Beaulieu and Gainetdinov 2011; Hall et al. 1994; Missale et al. 1998). Novel dopaminergic systems are still being described that are relevant to Parkinson’s disease—for example, thalamic dopaminergic pathways (Monje et al. 2020)—and it should be remembered that in terms of dopaminergic drugs, these act on all areas of the brain and their effects are not limited to the basal ganglia or to the brain since they are widely found in the peripheral nervous system (Amenta et al. 2002). Within the basal ganglia, dopamine receptors are classically portrayed as being present within the striatum and many of the concepts of the effect and side-effects of dopaminergic drugs in PD are wedded to this belief. But in fact, dopamine receptors are present in all major nuclei of the basal ganglia (caudate nucleus, putamen, globus pallidus, subthalamic nucleus) and all subtypes are present within each of these bodies with both presynaptic and post-synaptic localisations on a range of dopaminergic and non-dopaminergic neurones (Rommelfanger and Wichmann 2010). The function of most of these dopamine receptors is poorly understood and in particular, the function of those dopamine receptors located in non-striatal areas. In a similar way, dopamine innervation from the substantial nigra is normally portrayed as innervating the striatum. However, this is not correct as collaterals from the nigro-striatal pathway also innervate all other major basal ganglia nuclei and the thalamus (Hadipour-Niktarash et al. 2012; Lindvall and Bjorklund 1979; Rommelfanger and Wichmann 2010). These pathways also degenerate in PD, but little thought is given to the role they play in the expression of the symptomatology of the disease or indeed to the pattern in which these pathways degenerate over the course of the illness (Freeman et al. 2001).

Whilst the focus on dopamine agonists has been on those possessing D-2-like receptor activity, it is important to remember that both D-1 and D-2 receptors contribute to motor activity. D-1-like receptors are limited to a post-synaptic localisation whereas D-2-like receptors are found both on presynaptic terminals and on the post-synaptic membrane and both control dopaminergic transmission (Beaulieu and Gainetdinov 2011)—but this breaks down with the onset of PD. Similarly, in the normal brain, D-1 and D-2 receptors work in harmony to control motor function but in the denervated brain, D-1 and D-2 receptors work independently of one another (Arnt 1985; Arnt and Hyttel 1984). What this indicates is that stimulating both D-1 and D-2 receptors is a means to maximise motor activity from dopamine receptor stimulation. This might explain why currently used oral dopamine agonist drugs which are selective for D-2-like receptors appear in general to be of lower efficacy then levodopa as all receptors would be physiologically stimulated by dopamine in the normal brain. Attempts to selectively stimulate D-1-like receptors in PD and improve motor function have shown the efficacy of this approach in preclinical models of PD but have so far failed in man because of poor bioavailability and the onset of rapid tolerance due to D-1-like receptor downregulation—a phenomenon that may also affect D-2-like receptor stimulation in advanced PD (Kebabian et al. 1992; Shiosaki et al. 1996; Smith et al. 2002; Temlett et al. 1989).

## Apomorphine—just a dopamine agonist?

Apomorphine is the oldest drug used in the treatment of PD having a history going back thousands of years for its ritualistic use. As reviewed elsewhere, apomorphine was suggested for use in PD in the 1800’s, was forgotten until the early part of the 1900’s, revived in the 1960’s but only exploited in the 1980’s onwards when overcoming its lack of oral bioavailability was solved (Auffret et al. 2018a; Djamshidian and Poewe 2016; Kim et al. 2017). Its clinical efficacy by subcutaneous administration has been consistently demonstrated ever since but it is still underused (Castillo-Torres et al. 2023; Poewe and Wenning 2000). Apomorphine differs from the oral dopamine agonist drugs in that the quality of the motor response appears to be virtually indistinguishable from that of levodopa. In clinical studies, apomorphine produced greater than 90% of UPDRS response seen with levodopa and in the US pivotal study, apomorphine increased hand tapping speed to the same degree as levodopa (Dewey et al. 2001; Jenner and Katzenschlager 2016; Kempster et al. 1990). So, the question is why is apomorphine a better dopamine agonist than other drugs?

From a structural perspective, apomorphine is an ergoline that bears a strong resemblance to dopamine, but it is the receptor profile of apomorphine that may provide clues to its greater efficacy when compared to the oral non-ergoline dopamine agonists, ropinirole and pramipexole (Auffret et al. 2018a, 2018b, 2019; Jenner 1995, 2002; Jenner and Katzenschlager 2016; Kvernmo et al. 2006). The actions of the latter drugs are restricted to the D-2-like receptors whilst apomorphine interacts with both D-1-like and D-2-like receptor populations (De Keyser et al. 1995; Fici et al. 1997; Jenner 2002; Lam 2000; Lataste 1984). Apomorphine may also act to inhibit MAO-A and MAO-B and through this action potentiate the effects of dopamine and other monoamine neurotransmitters (Grunblatt et al. 1999).

This means that apomorphine has a broad-spectrum receptor profile which more closely resembles that of dopamine than observed with the other agonist compounds. It is worth noting that apomorphine does not show the selectivity for D-3 receptors shown by drugs such as pramipexole and this may explain, in part, why its use is associated with a low incidence of impulse control disorders (Barbosa et al. 2017). In addition, apomorphine interacts with a range of other monoaminergic receptors—adrenergic and serotoninergic receptors—again unlike the oral agonists which have a more restricted profile (Jenner and Katzenschlager 2016). This is clearly important as both noradrenergic and serotoninergic activity are disrupted in PD and contribute to both the motor and non-motor symptomatology of the illness (Lang and Obeso 2004). For example, the interaction of apomorphine with 5-HT2A receptors might explain the low incidence of visual hallucinations seen with the drug (Borgemeester et al. 2016).

To summarise, apomorphine interacts with all dopamine receptor subtypes and it is not just a dopamine agonist as it also alters noradrenergic and serotoninergic transmission. As a consequence, apomorphine acts on multiple neurotransmitter systems that are altered in PD and functions as a ‘multimodal drug’. Apomorphine may be a pioneer in this area of ‘dirty drug’ pharmacology, but the concept in itself is interesting as in PD we have shied away from ‘multimodal’ drugs in favour of highly focussed single action compounds leading to a situation where polypharmacy is commonly used to control the myriad of symptoms evident in the disease. This trend may be reversing as other ‘multimodal’ compounds are now being recognised as being of value in treating PD—for example amantadine, safinamide, and zonisamide (Murata 2010; Pagonabarraga et al. 2021; Rascol et al. 2021). An easy way to assess the value of apomorphine as a multimodal drug is to ask a pharmacologist because here apomorphine is viewed as the archetypal dopamine agonist drug, as evidenced by its prolific use in the pharmacological literature of the 1960–1990’s. Apomorphine is effective in all experimental models of PD—apomorphine-induced locomotor activity, apomorphine-induced stereotypy, apomorphine-induced climbing behaviour, apomorphine-induced circling behaviour (see for example Butcher and Anden 1969). In these scenarios, apomorphine is the drug of choice simply because it is a more reliable and effective pharmacological tool than the other more selective dopamine agonist drugs.

## Levodopa—a complex pharmacological conundrum

Now we come to what is probably the biggest conundrum in understanding the action of drugs in PD namely levodopa whose superior action has been unsurpassed in over 60 years (for a perspective on levodopa use see Fahn 2008, 2018; Gerlach et al. 2005; Lees et al. 2015; Olanow 2019; Olanow et al. 2008; Olanow and Stocchi 2018; Tolosa et al. 1998). Despite its undoubted success, levodopa would probably not be developed or approved today. It is not active itself being a prodrug, it lacks potency and a high dose is required. The drug has poor oral absorption, extensive metabolism, a short duration of effect and poor penetration into brain. It produces significant adverse events in man and insufficient toxicology of a modern standard was carried out in the era in which it was developed. For this day and age, insufficient placebo-controlled clinical trials have been undertaken that demonstrate its efficacy in PD (for an exception see Fahn 1999, 2006a, b). Had it not been for the development of peripheral decarboxylase inhibitors (which allowed for a reduction in dosage and the avoidance of peripheral side-effects), it is likely that levodopa would have been abandoned (Cotzias 1971). Similarly other types of enzyme inhibitors (COMTi, MAO-Bi) have been introduced to maximise the effects of each dose of levodopa by preventing its peripheral metabolism and that of dopamine produced from levodopa (see for example Nissinen and Mannisto 2010; Tan et al. 2022).

The general view is that levodopa acts through its conversion to dopamine and that dopamine then interacts with dopamine receptors. This reverses the dopamine deficiency that underpins PD. Certainly, the mainstay of that argument is correct, at least based on animal studies, but the dopamine derived from levodopa presumably interacts with all subtypes of dopamine receptors and as such produces a more physiological dopamine receptor stimulation that would be seen with the oral dopamine agonist drugs. In particular, it will, of course, stimulate both D-1-like and D-2-like receptors and from a pharmacological perspective, this would be expected to result in a more pronounced anti-parkinsonian effect than through stimulation of either individual receptor population. But even taking into account the short plasma half-life of the drug, the pulsatility of its effect and the changes which occur in its storage and buffering in dopaminergic neurones over the course of the disease, we struggle to understand the undoubted superiority of levodopa in treating symptoms of the illness compared to other pharmacological approaches and even to more modern technology-based therapies, such as deep brain stimulation.

However, the actions of levodopa are not restricted to its effects on the dopaminergic system and there is evidence of both direct and indirect noradrenaline and 5-HT involvement in levodopa’s actions—making it fit with the concept of a ‘multimodal drug’. Both noradrenergic and serotoninergic fibre pathways innervate the basal ganglia, they are involved in the control of motor function and both degenerate in PD (Lang and Obeso 2004). Although largely forgotten, there have been extensive studies of the effects of levodopa on noradrenergic transmission. Dopamine formed from levodopa is converted to noradrenaline by dopamine beta-hydroxylase and inhibition of noradrenaline synthesis decreases motor activity produced by levodopa in experimental models of PD (Dolphin et al. 1976). In a similar vein, serotoninergic neurones are implicated in the induction of levodopa-induced dyskinesia. Levodopa is decarboxylated to dopamine in serotoninergic neurones which is then released with serotonin in a non-physiological manner. This is why 5-HT1A or 5HT1B receptor agonists can suppress levodopa-induced dyskinesia—as these receptors control the firing of serotoninergic neurones and the release of neurotransmitter (Corsi et al. 2021; Pinna et al. 2023). The overall conclusion is that both noradrenaline and 5-HT are involved in the actions of levodopa and the control of motor function.

In addition to its effects on the dopaminergic, noradrenergic and serotoninergic systems, levodopa has been proposed as a neurotransmitter in its own right—although this is largely the view of one research group and this needs verification by others (see for review Misu and Goshima 1993). Levodopa fulfilled all the criteria for functioning as a neurotransmitter or neuromodulator. Levodopa immune-positive but aromatic amino acid decarboxylase immuno-negative neurones were identified in brain. Levodopa as the intact amino acid was shown to alter glutamate, acetylcholine and noradrenergic responses in the striatum. Motor activity to levodopa was reported to occur following inhibition of both central and peripheral dopa decarboxylase activity. If this view is correct, then we may have ignored something that is fundamentally important to the actions of this drug. However, one of our own studies is not in total agreement with these findings. We showed that blocking central dopa decarboxylase activity using NSD 1015 did not alter levodopa-induced rotation in 6-hydroxydopamine lesioned rats (Treseder et al. 2001)—suggesting an action of levodopa itself was responsible as previously proposed. But NSD 1015 treatment did not decrease striatal dopamine levels as might have been expected and appeared to reduce dopamine turnover. Since NSD 1015 can also act as an MAO-B inhibitor, it may have been this action which is responsible for the motor activity that was observed.

One further level of complexity needs to be considered as contributing to the response to levodopa in PD. There are two components to the drug’s activity—there is a short-duration response which in early disease is the immediate short-term effect seen after minutes to few hours after administration of each dose of levodopa and there is a long-duration response which takes days to weeks to become apparent (Albin and Leventhal 2017; Anderson and Nutt 2011; Nagao et al. 2019; Nutt et al. 2002). There is dispute over how disease progression impacts on the response to levodopa as far as these components are concerned. The long-duration response initially represents 30–50% of the total motor response but was reported to decline with disease progression (Stocchi et al. 2010). More recently, this view has been challenged and investigations of the effects of daily levodopa treatment on the progression of motor disability in overnight ‘off’ periods over 2 years have shown that the long-duration response persists independently of disease duration even in the most advanced stages of the illness (Cilia et al. 2020).

Whatever the truth, the long-duration response is clearly a key component of levodopa’s action. Whether the long-duration response also contributes to the actions of dopamine agonist drugs is less well studied and less clear (but see Barbato et al. 1997; Stocchi et al. 2001). This is good example of a ‘known unknown’ in understanding drug action in PD as the mechanisms responsible for the long-duration response are poorly understood and under investigated. It seems to involve some adaptive change in the motor response to levodopa, it could involve fundamental processes such as LTP/LDP (see for relevance to levodopa’s actions Calabresi et al. 2015), but nobody knows. Estimating the duration of any long-term response to levodopa represents a significant clinical challenge and changes in dopamine receptor sensitivity in response to more pulsatile or more continuous drug delivery may induce dynamic changes that affect its measurement (Cilia et al. 2020). It is not even clear whether the long-duration response is seen in the actions of the drug in experimental models of PD or whether it is a component of drug action restricted to the use in man, as the long-duration response was not observed in rat and monkey models, and they only presented the short-duration response and levodopa-induced dyskinesia (personal observation; Kuoppamaki et al. 2007)—this is a matter of debate. Solving this component of levodopa’s action might go a long way to developing novel more effective treatments for motor symptoms. This may not be through improving the bioavailability of levodopa as the short-duration response appears to reflect the plasma levels of levodopa, but the long-duration response is post-synaptic in nature and probably non-dopaminergic in origin (Barbato et al. 1997; Kuoppamaki et al. 2007).

## Levodopa—the ‘unknown unknowns’

Then we come to the parts of the action of levodopa and aspects of its mechanism of effect that are overlooked, forgotten or thought to be of little interest but which may represent key components of the drug’s action—and where ignoring the blindingly obvious may hinder the development of novel approaches to the treatment of PD. The examples given below are merely illustrations of perhaps how little we know about a drug which has been used routinely in PD for over 60 years. They centre around the metabolism of the drug (and even these do not take into account the potential generation of trace amines and other potential metabolites (for example, trihydroxyphenylalanine quinone and tetrahydroisoquinolines) that have been proposed as contributing to the actions and/or toxicity of levodopa (see for an example McNaught et al. 1998).

3-O-Methyldopa (3-OMD) is a major metabolite of levodopa in both the periphery and in brain (Nissinen and Mannisto 2010) but receives scant attention when considering the effects of levodopa. It is a terminal metabolite of the actions of catechol O-methyl transferase (COMT) that is not a substrate for dopa decarboxylase—and it is not metabolised further as far as we are aware although this may not have been investigated. A little-known fact is that peripheral inhibition of dopa decarboxylase, as is routinely used in the treatment of PD, diverts more levodopa into the catechol O-methyltransferase pathway and so elevates plasma levels of 3-0MD even further (Dingemanse et al. 1997). 3-OMD has a long plasma half-life (12–15 h) and as a consequence, accumulates in the periphery and in brain tissue. There is some evidence that 3-OMD may compete with levodopa for the active uptake process that transports the drug into brain, but this has not been extensively studied (Benetello et al. 1997). Whilst no adverse effects of 3-OMD have been demonstrated in man, how extensively this has been examined is not clear. Nor has the potential role that this compound plays in either the actions or adverse events of levodopa being fully evaluated or indeed, whether these are alleviated if levodopa is used in combination with a COMT inhibitor. Controversy exists over 3-OMD’s ability to reduce levodopa’s motor effects, the expression of dyskinesia and in the genesis of ‘wearing off’ (Fabbrini et al. 1987; Gervas et al. 1983; Nutt et al. 1987; Wade and Katzman 1975). There is some evidence to suggest that 3-OMD has the potential to inhibit locomotor activity, to decrease dopamine turnover, and to inhibit the dopamine transporter—but nothing sufficient to say that this occurs in man (Lee et al. 2008). At a pathogenic level, 3-OMD can induce oxidative stress, decrease mitochondrial membrane potential and potential cell death, all of which are thought to be components of cell death in Parkinson’s disease—at least in preclinical studies. It may be that 3-OMD is completely inert and a diversion but until we evaluate its role in the actions of levodopa and its role in its own right, we will not know the role played by this major metabolite of the most commonly used drug in PD.

There are further examples which illustrate either lack of knowledge or a failure to understand other events linked to the metabolism of levodopa of relevance to PD. Only recently, have potentially important changes in peripheral dopa decarboxylase activity been uncovered—and in all probability, most people are unaware of these. In three independent cohorts of patients with PD or parkinsonism on levodopa plus a decarboxylase inhibitor, elevated levels of dopa decarboxylase (L-aromatic amino acid decarboxylase) enzyme activity were present in 82% of patients in this population (van Rumund et al. 2021). Those patients with elevated enzyme activity had a longer disease duration and were on higher doses of levodopa leading to the suggestion that these changes might contribute to the decrease in levodopa effectiveness and the need for higher doses with disease progression. But, so far, this is merely an observation and the mechanism underlying the increase in decarboxylase activity, and its clinical consequences remain unknown. In a similar manner, alterations in the gut microbiome leading to changes in the decarboxylation of levodopa by bacterial tyrosine decarboxylases alter the bioavailability of levodopa and its effectiveness in PD (Maini Rekdal et al. 2019)—and this also requires further investigation as these enzymes are not blocked by the peripheral decarboxylase inhibitors used in PD.

One final example illustrates our failure to question the actions of compounds routinely used in the treatment of PD. We tend to accept as fact the classical definitions of their activity and this becomes truth by repetition. A good example is again looking at the peripheral decarboxylase inhibitors where it is commonly accepted that these do not penetrate into brain—but this is not entirely true. They are selective inhibitors of the peripheral enzyme but not specific in their actions and at higher doses may affect brain decarboxylase activity—which would prevent dopamine formation. In fact, there has been very little examination of the specificity of the decarboxylase inhibitors in general—as they were developed in an era where this was not routinely undertaken. For example, in a study that we undertook, we showed that both carbidopa and benserazide blocked central MAO-B activity (Treseder et al. 2003). One interpretation of this would be that it complicates how we think about the role in potentiating of the decarboxylase inhibitors in potentiating the effects of levodopa. Whilst we continue to use some of the older tools in the toolbox without question, it could be that what we are doing is adopting a blinkered approach that stops progress.

All of this raises the question of whether we really understand how levodopa’s actions are manifest in PD. This is of fundamental significance as everything we strive to achieve currently is based on the simplistic view that levodopa yields dopamine that then interacts with striatal dopamine receptors and that is what we should try to emulate in devising new approaches to treatment.

## Is there a way forward?

The conclusion of all this is that perhaps Donald Rumsfeld is right even though he received a degree of ridicule when he gave this particular statement. Perhaps we do not understand precisely how established drugs for the treatment of PD produced their clinical effects, but do we question why drugs like levodopa and apomorphine appear more effective than others? Should we look beyond the dopamine system for answers? We need to reinvest pharmacological studies and conduct again “basic” studies to better understand how these drugs work. In recent times, there has been a lack of sound studies exploring the pharmacokinetics and pharmacodynamics of these drugs in humans—and very few patients with PD ever have their plasma drug profiles measured. There are probably ‘unknown unknowns’ that we chose to ignore. Poking life in the eye with a sharp stick is often a productive way of making progress.

## Data Availability

The data included in this review is freely available from the normal literature sources and has been derived from PubMed searches.
